# Health disparities in COVID-19: immune and vascular changes are linked to disease severity and persist in a high-risk population in Riverside County, California

**DOI:** 10.1186/s12889-023-16462-5

**Published:** 2023-08-19

**Authors:** Kristina V. Bergersen, Kathy Pham, Jiang Li, Michael T. Ulrich, Patrick Merrill, Yuxin He, Sumaya Alaama, Xinru Qiu, Indira S. Harahap-Carrillo, Keita Ichii, Shyleen Frost, Marcus Kaul, Adam Godzik, Erica C. Heinrich, Meera G. Nair

**Affiliations:** 1 Division of Biomedical Sciences, School of Medicine, University of California Riverside, Riverside, CA U.S.; 2 Riverside University Health System Medical Center, Riverside, CA U.S.; 3 Kaiser Permanente Riverside Medical Center, Riverside, CA U.S.

**Keywords:** Health disparities, Hispanic, Immunology, COVID-19, Severe infection, Long COVID

## Abstract

**Background:**

Health disparities in underserved communities, such as inadequate healthcare access, impact COVID-19 disease outcomes. These disparities are evident in Hispanic populations nationwide, with disproportionately high infection and mortality rates. Furthermore, infected individuals can develop long COVID with sustained impacts on quality of life. The goal of this study was to identify immune and endothelial factors that are associated with COVID-19 outcomes in Riverside County, a high-risk and predominantly Hispanic community, and investigate the long-term impacts of COVID-19 infection.

**Methods:**

112 participants in Riverside County, California, were recruited according to the following criteria: healthy control (*n* = 23), outpatients with moderate infection (outpatient, *n* = 33), ICU patients with severe infection (hospitalized, *n* = 33), and individuals recovered from moderate infection (*n* = 23). Differences in outcomes between Hispanic and non-Hispanic individuals and presence/absence of co-morbidities were evaluated. Circulating immune and vascular biomarkers were measured by ELISA, multiplex analyte assays, and flow cytometry. Follow-up assessments for long COVID, lung health, and immune and vascular changes were conducted after recovery (*n* = 23) including paired analyses of the same participants.

**Results:**

Compared to uninfected controls, the severe infection group had a higher proportion of Hispanic individuals (*n* = 23, *p* = 0.012) than moderate infection (*n* = 8, *p* = 0.550). Disease severity was associated with changes in innate monocytes and neutrophils, lymphopenia, disrupted cytokine production (increased IL-8 and IP-10/CXCL10 but reduced IFNλ2/3 and IFNγ), and increased endothelial injury (myoglobin, VCAM-1). In the severe infection group, a machine learning model identified LCN2/NGAL, IL-6, and monocyte activation as parameters associated with fatality while anti-coagulant therapy was associated with survival. Recovery from moderate COVID infection resulted in long-term immune changes including increased monocytes/lymphocytes and decreased neutrophils and endothelial markers. This group had a lower proportion of co-morbidities (*n* = 8, *p* = 1.0) but still reported symptoms associated with long COVID despite recovered pulmonary function.

**Conclusion:**

This study indicates increased severity of COVID-19 infection in Hispanic individuals of Riverside County, California. Infection resulted in immunological and vascular changes and long COVID symptoms that were sustained for up to 11 months, however, lung volume and airflow resistance was recovered. Given the immune and behavioral impacts of long COVID, the potential for increased susceptibility to infections and decreased quality of life in high-risk populations warrants further investigation.

**Supplementary Information:**

The online version contains supplementary material available at 10.1186/s12889-023-16462-5.

## Introduction

The United States alone currently accounts for 103 million of the 760 million confirmed COVID-19 cases world-wide, and 1.1 million confirmed deaths have been reported in the US as of June 2023 [1]. Of the 66 million reported cases with ethnicity data available, Hispanic individuals make up 24% of these cases despite only making up 18.45% of the total US population [2]. The Hispanic population also comprises 17% of all reported deaths, one of the largest groups impacted by infection [2]. Despite a decreasing trend observed in COVID-19 hospitalizations and deaths, and significant advances in vaccine development and distribution in the last two years, the proportion of COVID-19 positive cases and deaths in the Hispanic community remains high. Possible reasons include existing health disparities like pre-existing health conditions and higher rates of key co-morbidities [3–6]. A higher prevalence of these pre-existing factors is predicted to stem from systemic inequities in healthcare access, quality of care, and inequalities in the built environment. In the county of Riverside, California, Hispanic individuals make up 37% of the cumulative 741,000 reported COVID cases, almost twofold more than reported cases in non-Hispanic white individuals [7]. In addition, Hispanic individuals make up 42% of the hospitalizations and deaths in this county. This staggering statistic is concerning given the lack of Hispanic data in COVID-19 research.

COVID-19 infection induces significant immune and vascular responses that change with disease severity. Severe infection often results in overactivation of certain innate immune subsets [8–15] while additional studies have reported suppression of adaptive immune subsets that are necessary for antiviral immunity and memory responses [16–18]. These highly dysregulated immune responses not only represent their own form of infection-induced pathology but also drive vascular pathology, including coagulopathies, myocarditis, and tissue damage particularly in the lung following infection [10, 19, 20]. Immune-independent mechanisms underlying COVID-19-induced vascular damage, including changes in essential vitamins and platelet aggregation, have also been reported [21]. Dysregulated immune responses may therefore play a larger role in COVID-19-induced vascular pathology than originally thought.

Increasing reports of post-acute COVID-19 conditions raise serious concerns for unknown long-term effects of this disease. These conditions are similar to other post-acute infection syndromes that have long been present but remain widely misunderstood [22]. Long COVID is characterized by persistent symptoms, immune dysregulation, and lasting tissue damage that persists after recovery from infection [23–26]. Recent work has identified a relationship between COVID-19 disease severity and the development of long COVID [27]. Prolonged immune changes associated with long COVID are present for months following recovery in the periphery [28, 29] as well as in tissues directly affected by infection [30]. Measures have been taken to evaluate and manage long COVID based on current knowledge [31], but significant gaps in knowledge remain about the risk factors and underlying mechanisms [32].

The primary goal of this study was to identify immune and endothelial biomarkers associated with disease severity in Riverside County, CA. Additionally, a machine learning model was used to dissect large data output and predict risk factors for COVID-19 disease severity. A secondary goal was to determine if Hispanic individuals were disproportionately affected by COVID-19 infection. Finally, a third goal of this study was to explore immune and vascular factors associated with long COVID in this cohort and investigate the impact on lung health and immune homeostasis.

## Materials and methods

### Study population

Men and women with active COVID-19 infection, who had recovered from a past mild-to-moderate COVID-19 infection, or had no history of COVID-19 (healthy control) were recruited. Individuals who were currently incarcerated or were not residents of Riverside County, CA, were excluded, as were individuals under the consenting age of 18. Pregnant women were excluded from the study due to exposure to acute hypoxia and hypercapnia in breathing tests not reported here. Severe group participants who received treatment (e.g., dexamethasone) prior to sampling were not excluded. Inclusion criteria for each study group were: 1) healthy controls with no prior history of confirmed COVID-19 infection (via PCR or rapid test); 2) moderate infection participants that were outpatients with active COVID-19 infection (confirmed by PCR test), who were offered monoclonal antibody treatment; 3) severe infection participants with active COVID-19 infection (confirmed by PCR test) requiring hospitalization and supplemental oxygen; 4) recovered participants with no active COVID-19 infection but prior history of confirmed positive (via PCR or rapid test) mild-to-moderate COVID-19 infection.

### Study recruitment and design

#### Study recruitment

Moderate infection participants were invited to participate by fliers distributed by their physician. Severe infection participants were recruited from Riverside University Health System (RUHS). For Control and Recovered groups, participants were recruited from University of California Riverside Health Clinic, Riverside Free Clinic, and University of California, Riverside.

#### Study design

Participants completed a medical history questionnaire which provided information regarding demographic information, current medications, as well as current and past medical conditions. Recovered participants also completed the Yorkshire Rehabilitation Scale questionnaire which provides self-reported health status before and after recovery from COVID-19 [33, 34]. For recovered participants and healthy controls, basic physiological parameters including height, weight, blood pressure, and lung function parameters via spirometry were recorded. Peripheral venous blood samples were collected from all participants using standard phlebotomy procedures. For ICU patients, data was collected from hospital medical records, hospital personnel drew blood, and specimens were transported to the University of California, Riverside for processing. For all groups, samples were processed for downstream analysis within 4 h of collection. Samples were collected between January 2021-February 2022 from RUHS for the severe (hospitalized) group, January-July 2022 for the moderate (outpatient) group, April–October 2022 for healthy control group, and April–October 2022 for follow-up (recovered) group.

### Laboratory evaluation of samples

#### Whole blood and plasma isolation

Peripheral venous blood was collected in 10 ml vacutainer tubes containing EDTA (BD, Franklin Lakes, NJ, USA) and maintained at room temperature until processing within 4 h of collection. Following blood collection, an aliquot of whole blood was set aside for flow cytometry analysis. Plasma was isolated according to previously published protocols [35]. Briefly, gradient centrifugation with Histopaque-1077 was performed, and plasma was recovered for quantification of cytokines, Resistin, and endothelial parameters.

#### Flow cytometry

100 µl whole blood was incubated in human Fc Receptor Block (Biolegend) followed by anti-human fluorophore-conjugated antibodies: PerCP/Cyanine5.5 anti-CD3), APC/Cyanine7 anti-CD11b, Brilliant Violet 650 anti-CD25, PE/Cyanine5 anti-CD62P/P-Selectin, Brilliant Violet 711 anti-CD45, APC anti-CD66b, Brilliant Violet 605 anti-CD56/NCAM, Alexa Fluor488 anti-CD14, Brilliant Violet 785 anti-CD8, PE anti-CD16 (all from Biolegend); Alexa Fluor594 anti-ACE-2 (R&D Systems), PerCP-eFluor710 anti-CD19 (eBioscience), and BV421 anti-HLA-DR, DP, DQ (BD Biosciences). Red blood cells were lysed and cells were fixed using 1-step Fix/Lyse Solution (eBioscience), rinsed with FACS buffer, and resuspended in FACS buffer (0.5% BSA, 0.005% EDTA, 1 × PBS). Samples were analyzed using the NovoCyte Quanteon flow cytometer, NovoSampler Q, and NovoExpress Software. An average of 1 × 10^5^ events were collected from the “Live Cell” gate for analysis. Analysis was conducted using FlowJo software version 10. The complete gating strategy is provided in Supplemental Fig. 2.

#### Participant-specific immune tracking

Within each study group, some participants (Moderate, *n* = 3; Severe, *n* = 9; Control/Recovered, *n* = 5) provided blood specimens at multiple timepoints. These samples were used for within-subject time course analyses to determine changes in parameters throughout the course of infection and following recovery. Flow cytometry for different immune cell populations was analyzed for each of these participants to track changes over time. A representative participant was then selected for each group, and UMAP projections were created to visualize immune cell changes.

#### Cytokine and endothelial marker analysis

Plasma samples were assayed for cytokine and endothelial marker analysis using LEGENDplex kits and the manufacturer’s protocols (Biolegend): “Human Anti-Virus Response Panel” (13-plex) at 1:2 dilution and “Human Vascular Inflammation Panel” (13-plex)”at 1:100 dilution in 5% TX100 (in PBS) for virus inactivation. Samples were run on a Novocyte Quanteon flow cytometer, and data analysis was conducted using BioLegend’s LEGENDplex Data Analysis Software.

#### ELISA

The ELISA assays for Resistin and spike protein receptor-binding domain (RBD) were performed with the following ELISA kits: Human-Resistin-Mini ABTS ELISA Development Kit (PEPRO TECH, Catalog #900-M235). Human SARS-CoV-2 RBD ELISA Kit (Themo Fisher, Catalog # EH492RB). Prior to measurement, the plasma samples were centrifuged at 1000 g for 5 min, supernatant was then diluted 1:100 for Resistin and 1:4 for spike protein. The absorbance was acquired by a plate reader (BioTek Synergy HT).

#### Viral reverse transcription quantitative PCR

RNA was extracted from nasal swab samples using a Quick-RNA Viral Kit (ZYMO Research, Catalog # R1034). QPCR was performed using primers (N2) and probes from the 2019-nCoV RUO Kit (IDT, Catalog # 10,006,713). SARS-CoV-2 RNA was quantified using GoTaq® Probe 1-Step RT-qPCR System (Promega, A6120) according to the manufacturer’s protocol. 1000 copies/µL of 2019-nCoV plasmid (IDT, Catalog # 10,006,625) was used as positive control.

#### Lung function spirometry tests

To measure lung function in recovered participants and healthy controls, standard spirometry procedures were conducted in triplicate and mean values were collected for forced vital capacity (FVC), forced expired volume in 1 s (FEV_1_), and FEV_1_/FVC. Values were adjusted for predicted values based on the National Health and Nutrition Examination Survey (NHANES) III reference values, or Global Lung Function Initiative (GLI) references values for Asian individuals. Respiratory flow was measured with a spirometer and respiratory flow head (1000 L) (ADInstruments, Colorado Springs, CO, 80,907), with data acquisition via a PowerLab 8/35 and analysis conducted in LabChart Pro using the ADInstruments spirometry plugin.

### Statistical and bioinformatic analysis

#### Statistical analysis for laboratory results

Statistical significance for all experiments was determined by either 2- tailed unpaired Student’s t-test or One-Way ANOVA with multiple comparisons and a *p*-value < 0.05 was considered statistically significant. For self-reported symptom severity on the YRS Questionnaire, data was tested for normality of distributions with Shapiro Wilk’s tests, and scores were compared across pre and post COVID-19 infection using paired Wilcoxon-signed rank tests in R using the *stats* package. *P*-values for these comparisons were adjusted for family-wise error rates using a Holm-Bonferroni method. The type of statistical test run for each experimental result is indicated in the corresponding figure and table legends.

#### Correlation analyses

To examine correlations between parameters in the dataset, pairwise Pearson correlations were conducted using the R package *Hmisc*. Using the min–max scaling method, all continuous variables were scaled from 0 to 1 to guarantee that they were all on the same scale. Binary variables were converted to 1 (yes) or 0 (no). Correlation analysis results were visualized using the *corrplot* function in R. Correlation coefficients with *p*-values less than 0.05, 0.01, and 0.001 are denoted with “*,” “**,” and “***” respectively.

#### Machine learning (ML)

We trained the model using three sets of data: one set of parameters acquired from the hospital, one set of parameters lab-based assays, and one set with both types of parameters. The parameters from the hospital included 17 features “Sex”, “Hispanic”, “Diabetes”, “Hypertension”, “SBP”, “DBP”, “RBCs”, “WBC”, “Platelets”, “AST”, “ALT”, “LDH”, “CRP”, “Ferritin”, “D-dimer”, “HgbA1C”, “Anticoagulant”. The parameters from lab-based assays included 19 features including “Platelets Percentage of Whole Blood”, “Neutrophils”, “Monocytes”, “B cells”, “MHCII Monocytes”, “Resistin”, “IL-8”, “IP-10”, “IL-6”, “IFNλ2/3”, “MHCII + Platelets”, “LCN2/NGAL”, “Myoglobin”, “CRP”, “OPN”, “MPO”, “ICAM-1”, “VCAM-1”, “Cystatin C”. Prior to training the ML models, the data was pre-processed by addressing missing values. For the missing values, the average value was used for imputation. In the present study, missing values of HgbA1C were imputed using a HgbA1C threshold approach. Specifically, for non-diabetic participants, missing values were filled with a value of 5.7, which is the lower limit of the normal HgbA1C range. For diabetic participants, missing values were filled with a value of 6.5, which is the diagnostic cut-off for diabetes. For the undetected values from ‘IL-6’, ‘IFN’, and ‘Cystatin C’, half of the lowest measurable value was used to impute. Random forest was applied from the scikit-learn library to train the data. The ‘leave-one-out’ cross-validation was used to evaluate the performance of the model and the feature_importances function provided by the random forest to select the features as the best combination predictors. In random forest, feature importance is computed by averaging the importance of a feature across all trees in the forest. The feature importance score is calculated as the average decrease in impurity (measured by Gini impurity) caused by splits involving the feature. These selected features were then utilized to train a Simple Decision Tree Classifier. A visualization of the decision tree generated from this classifier was created to provide an intuitive representation of the relationships between the features and the outcome.

## Results

### Hispanic individuals and co-morbidities are over-represented in severe infection

**Fig. 1:**
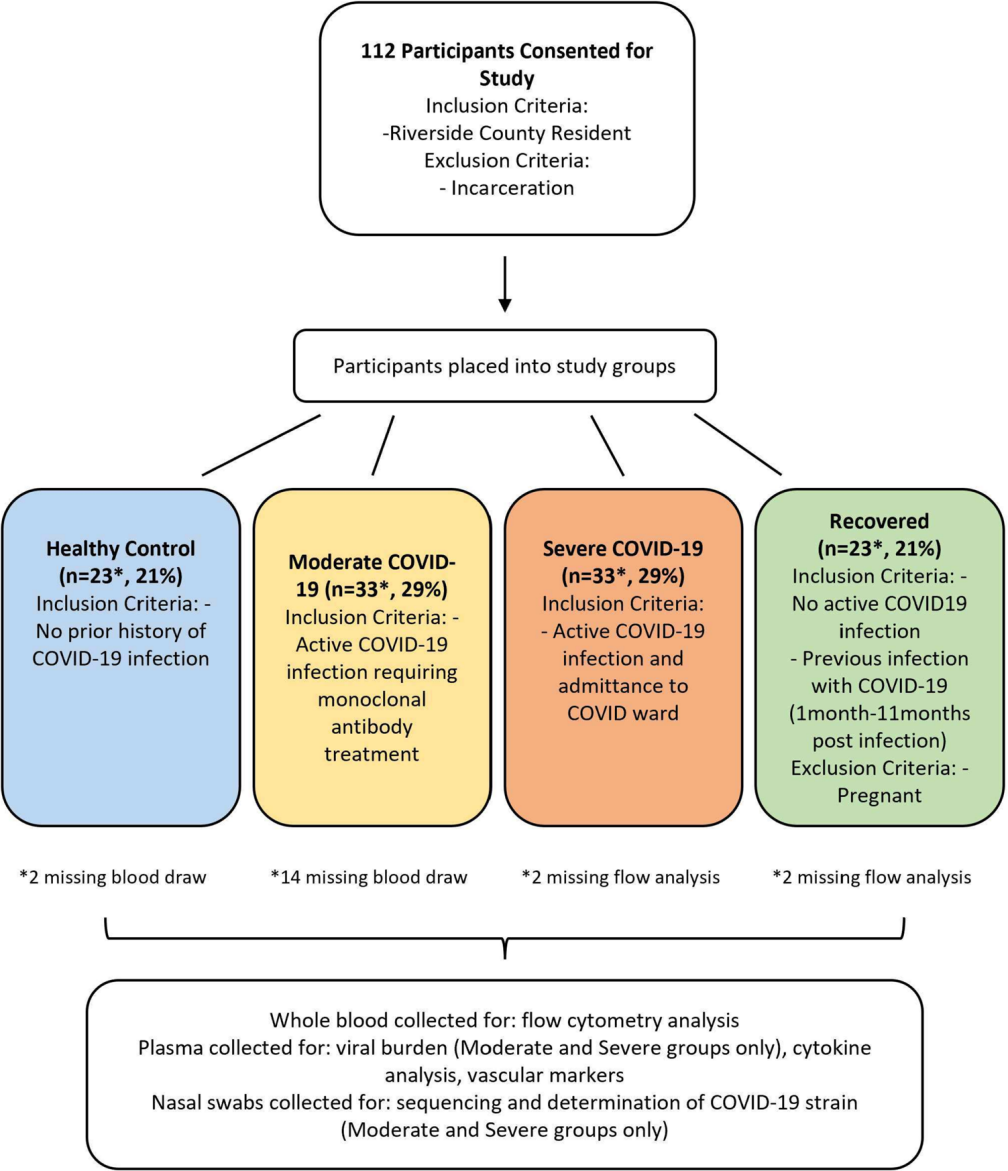
Flow diagram of study design, subject enrollment, grouping criteria and experimental methods

A total of 112 participants were included in this study and were placed into groups based on their COVID-19 status: 1) healthy control (*n* = 23), 2) outpatients with moderate COVID-19 infection (*n* = 33), 3) ICU patients with severe COVID-19 infection (*n* = 33), and 4) recovered (*n* = 23) (Fig. 1). Baseline characteristics are shown in Table 1. *P*-values indicate results of comparisons between each experimental group and healthy control group for each listed characteristic. Participant ethnicity data is based on self-reporting. Non-Hispanic group included individuals self-reporting as White (not of Hispanic origin), Asian or Pacific Islander, Black (not of Hispanic origin), or multiracial. The number of male and female participants were balanced between each experimental group compared to healthy controls. The mean age of moderate and severe infection groups was balanced (*p* = 0.398), and recovered and healthy control groups had comparable mean ages (*p* = 0.270). Active infection groups had increased age compared to healthy controls (*p* < 0.001). Hispanic individuals were significantly over-represented in the severe infection group (69.7%, *p* = 0.012) compared to other groups and the demographic data for Riverside County (51.6% Hispanic) [36]. There were also significantly more co-morbidities, including diabetes and hypertension, reported in the severe (*p* < 0.001) and moderate (*p* = 0.002) infection groups compared to healthy controls. Vaccination status also differed significantly in moderate (*p* = 0.001) and severe (*p* < 0.001) infection groups compared to healthy controls while the recovered group had comparable vaccination status (*p* = 0.480). This difference may be partially explained by differences in vaccine availability during sample collection from infected individuals compared to healthy participant recruitment. Overall, these data indicate that Hispanic individuals and reported co-morbidities were over-represented in the severe infection group which correlates with hospitalization and death statistics in Riverside County for the time period of the study (2021–2022) [7].

**Table 1:** Study demographics by group

Characteristic	Healthy Control *n* = 23(%)	Outpatient Moderate Infection *n* = 33(%)	*P*-value	ICU Severe Infection *n* = 33(%)	*P*-value	Recovered *n* = 23(%)	*P*-value
**Sex**							
**Male**	12 (57)	13 (39)	0.27	23 (70)	0.39	10(48)	0.76
**Female**	9 (43)	20 (61)		10 (30)		11(52)	
**Age, years**							
**Mean (SD)**	27.60 (7.27)	52.88 (16.68)	**0.0003**	56.09 (13.86)	** < 0.001**	31.05 (12.17)	0.27
**Median (IQR)**	26.00 (19.00–52.00)	48.00 (22.00–84.00)		52.00 (23.00–80.00)		27.00 (19.00–67.00)	
**Ethnicity, No. (%)**							
**Hispanic**	7 (33)	8 (25)	0.55	23 (70)	**0.012**	9 (43)	0.75
**Non-Hispanic**	14 (67)	24 (75)		10 (30)		12( 57)	
**Co-morbidity, No. (%)**							
**Yes**	8 (38)	27 (82)	**0.002**	30 (91)	** < < 0.001**	8 (38)	1
**No**	13 (62)	6 (18)		3 (9)		13 (62)	
**Vaccinated, No. (%)**							
**Yes**	18 (95)	10 (45)	**0.001**	9 (28)	** < < 0.001**	21 (100)	0.48
**No**	1 (5)	12 (55)		23 (72)		0 (0)	

*P-values indicate results of comparisons between each experimental group and healthy control group for each listed characteristic. P-values generated using One-Way ANOVA with adjustments for multiple comparisons*

### Confirmation of COVID-19 viral burden in moderate and severe infection

To confirm active COVID-19 infection and determine viral demographics, nasal viral burden and circulating spike protein in plasma were evaluated in moderate and severe infection groups. Participants with moderate and severe infection had detectable levels of nasal viral burden with significantly higher viral load in the severe infection group (Supplemental Fig. 1A). Circulating spike protein concentrations were also compared across mortality outcomes within the severe infection group, and there was no significant difference between COVID-19 survivors or non-survivors (Supplemental Fig. 1B).

### Immune cell subsets and circulating immune and endothelial factors are dependent on COVID-19 disease severity

**Fig. 2:**
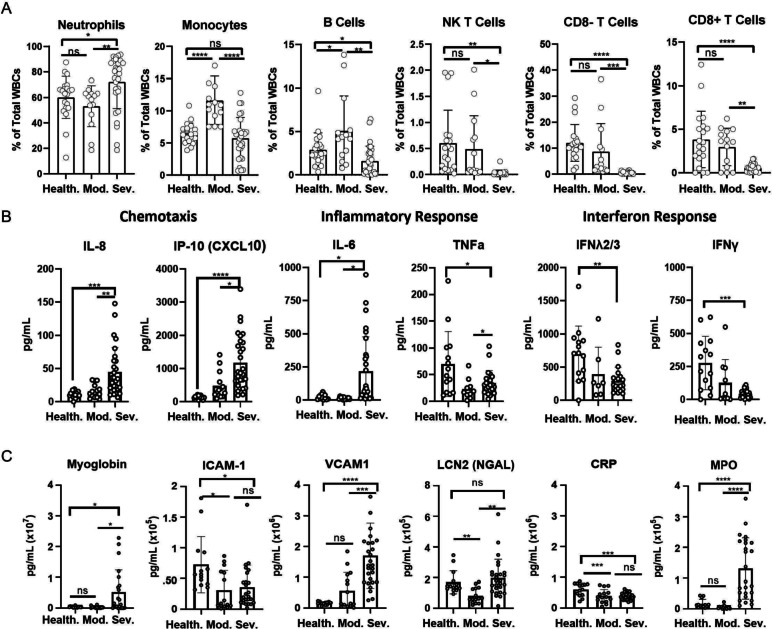
Immune and endothelial damage analysis during active COVID-19 infection. **A** Quantification of significantly altered immune populations from analyzed flow cytometry data. **B** Circulating cytokine levels from plasma of control and infected groups. **C** Circulating endothelial damage markers from plasma of control and infected groups. For all experiments, “Healthy” *n* = 21, “Outpatient/Moderate” *n* = 19, “ICU/Severe” *n* = 31, “n” altered for some results depending on availability of samples. Statistical significance determined via One-Way ANOVA using multiple comparisons

Immune cell subsets and plasma factors were measured to identify infection-induced changes and associations with disease severity. Flow cytometry gating of the peripheral blood was performed to quantify innate and adaptive immune cells and platelets (Fig. 2A-C, Supplemental Fig. 2). Flow plots of major immune populations from concatenated data for each study group demonstrated differences in key immune cell subsets (Supplemental Fig. 3). In severe infection, innate neutrophils were significantly elevated compared to moderate infection (*p* = 0.010) and healthy controls (*p* = 0.031), and monocytes were significantly decreased compared to moderate infection (*p* < 0.0001). B cells (*p* = 0.015), NK T cells (*p* = 0.002), CD8- T cells (*p* = 0.0002), and CD8 + T cells (*p* < 0.0001) were all significantly decreased in the severe infection group compared to healthy controls and moderate infection (B cells: *p* = 0.002; NK T cells: *p* = 0.020; CD8- T cells: *p* = 0.030; CD8 + T cells: *p* = 0.009) (Fig. 2A). These results demonstrate alterations in both innate and adaptive immune subsets that are dependent on disease severity.

**Fig. 3:**
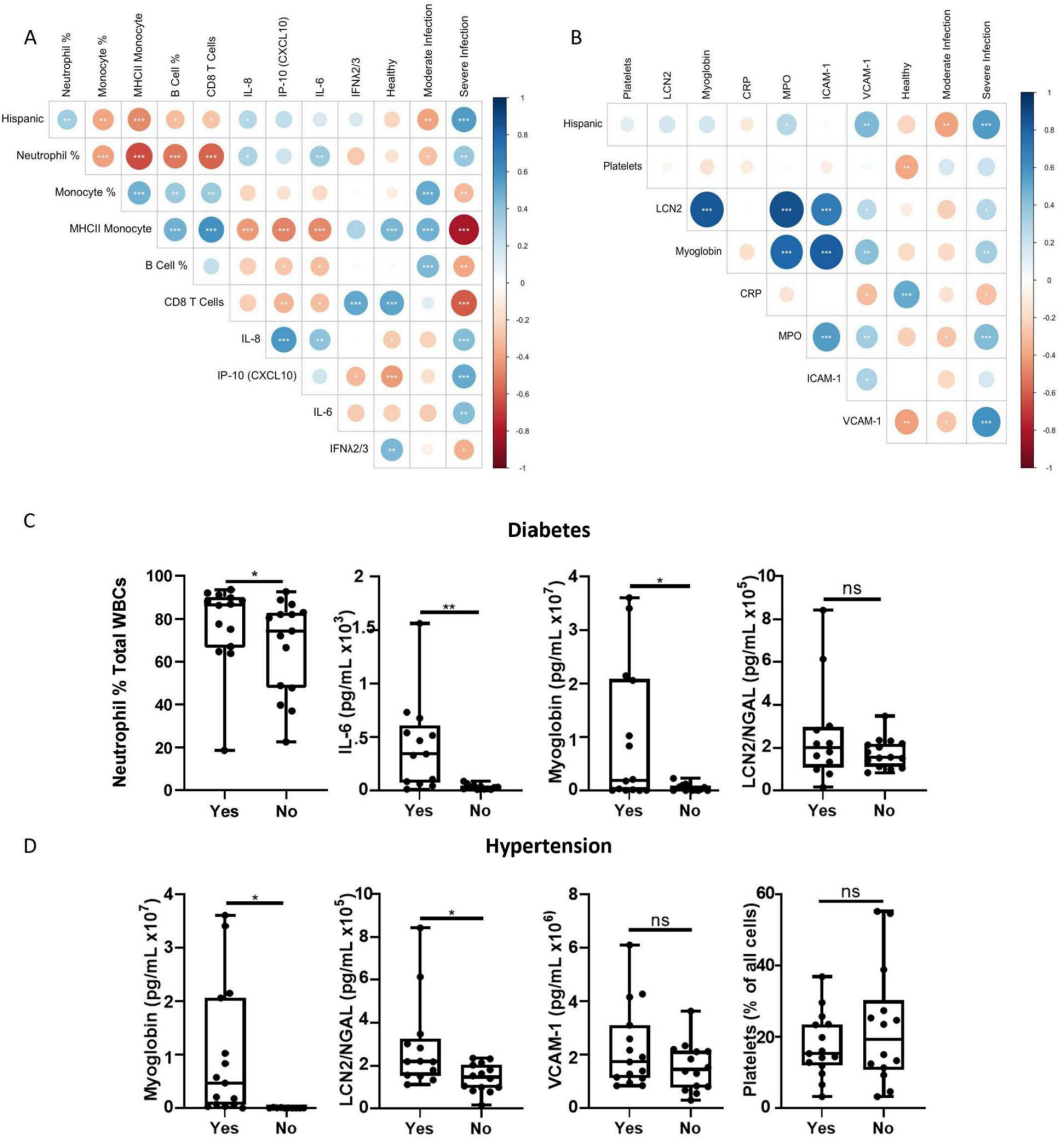
Correlation analyses of immune, endothelial, and clinical parameters. **A**-**B** Correlation matrix of immune (control *n* = 21, outpatient/moderate infection *n* = 19, ICU/severe infection *n* = 31) (**A**) and endothelial (control *n* = 15, moderate infection *n* = 19, severe infection *n* = 31) (**B**) parameters across control and infected groups. **C**-**D** Circulating levels of immune and endothelial parameters that have significant correlations with the clinical parameters of diabetes (**C**) and hypertension (**D**) in the severe COVID-19 group (*n* = 29). Statistical significance determined via unpaired student’s t-test

Significant changes were observed in circulating chemokines and interferons relating to chemotaxis, inflammatory, and anti-viral responses in severe infection (Fig. 2B). The neutrophil chemoattractant IL-8 was significantly elevated compared to both healthy controls (*p* = 0.0004) and moderate infection (*p* = 0.002). The chemoattractant IP-10 (CXCL10) was also significantly increased compared to healthy controls (*p* < 0.0001) and moderate infection (*p* = 0.002). Pro-inflammatory IL-6 was significantly increased in severe infection (*p* = 0.009) while TNFα was decreased (*p* = 0.016) compared to healthy controls. Anti-viral IFNλ2/3 (*p* = 0.002) and IFNγ (*p* = 0.0003) were also significantly decreased in severe infection.

Indicators of endothelial damage were also altered based on disease severity (Fig. 2C). Myoglobin (*p* = 0.02), VCAM1 (*p* < 0.0001), and myeloperoxidase (MPO) (*p* < 0.0001) were all significantly upregulated in the plasma of the severe infection group compared to healthy controls. There were also multiple endothelial markers that that were downregulated in severe infection including ICAM-1 (*p* = 0.02) and C-reactive protein (CRP) (*p* = 0.0003). Lipocalin (LCN)-2 (NGAL) was not significantly altered in severe infection (*p* = 0.77) but was significantly decreased in the moderate infection group (*p* = 0.001). Collectively, this data demonstrates severity-dependent changes in immune populations, cytokine response, and endothelial damage.

### Immune and endothelial biomarkers are impacted by the presence of co-morbidities during severe infection and are associated with fatal outcomes

To discern potential correlations between Hispanic ethnicity, infection severity, and immune/endothelial changes, correlation matrices were plotted for immune and endothelial parameters (Fig. 3). Hispanic individuals showed strong positive correlations with severe infection (Fig. 3A, B). Hispanic ethnicity was also positively correlated with elevated neutrophils and IL-8 but negatively correlated with monocytes, MHC II expression by monocytes, B cells, and CD8 + T cells (Fig. 3A). Analysis of endothelial parameters revealed that Hispanic ethnicity was positively correlated with VCAM-1 and MPO (Fig. 3B) which corresponds to elevated levels of these markers seen in severe infection (Fig. 2).

The clinical co-morbidities diabetes and hypertension that were over-reported in the severe infection group showed significant impacts on immune and endothelial parameters that were associated with severe disease (Fig. 3C, D). Participants with diabetes had increased circulating neutrophils (*p* = 0.04), IL-6 (*p* = 0.004) and myoglobin (*p* = 0.02) but no differences in circulating LCN2/NGAL (p = 0.19) (Fig. 3C). Participants with hypertensions had increased circulating myoglobin (*p* = 0.02) and LCN2/NGAL (*p* = 0.02), but no changes in VCAM-1 (*p* = 0.15) or platelets (*p* = 0.30) (Fig. 3D). These results demonstrate that both immune and endothelial parameters are correlated with disease severity and are altered based on the presence of co-morbidities that may reflect health disparities in the severe infection group.

To investigate relationships between demographic, immune, and endothelial parameters based on the outcome of severe infection, correlation matrices were performed for survival vs. fatality outcomes (Supplemental Fig. 4). Severely infected participants with a fatal outcome had more significant correlations between these parameters (Supplemental Fig. 4A) compared to those that survived (Supplemental Fig. 4B). In fatal infection outcomes, there were significantly negative correlations between Hispanic ethnicity and the cytokine Resistin, but there were no strong positive correlations between Hispanic ethnicity and any other parameter (Supplemental Fig. 4A). In the survival group, Hispanic ethnicity was not significantly correlated to Resistin or IL-6 but exhibited negative correlations with the endothelial parameter Cystatin C (Supplemental Fig. 4B).

**Fig. 4:**
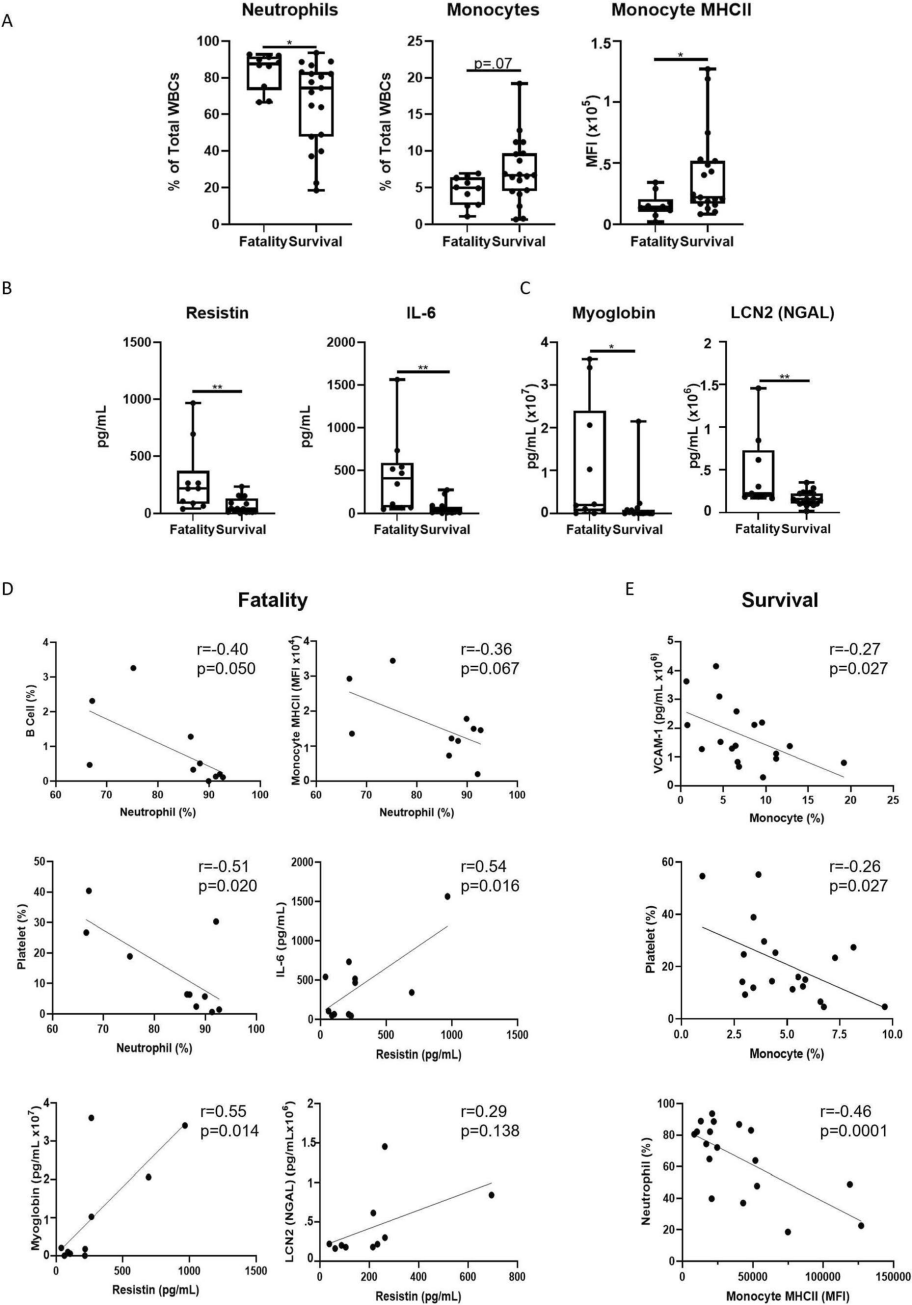
Analysis of immune and endothelial factors during severe COVID-19 infection based on infection outcome. **A** Significantly altered immune populations from flow cytometry data distinguished by fatality or survival. **B** Significantly altered circulating cytokine levels from fatal vs. survival groups. **C** Significantly altered circulating endothelial damage markers from plasma of fatal vs. survival groups. **D**-**E** Correlations between parameters within fatal (**D**) and survival (**E**) groups. Statistical significance determined via unpaired student’s t-test

The factors that were differentially correlated in fatal vs. survival outcomes were investigated further to determine if these differences contributed to a fatality (Fig. 4). Neutrophils were significantly increased in the fatal (*p* = 0.03) compared to survival group (Fig. 4A). Expression of MHCII by monocytes was significantly downregulated in the fatal group (*p* = 0.04) despite there being no significant changes in monocyte percentages (*p* = 0.07) (Fig. 4A). The fatal group also had significantly increased plasma Resistin (p = 0.008) and IL-6 (*p* = 0.003) compared to the survival group (Fig. 4B). Finally, the endothelial damage indicators myoglobin (*p* = 0.01) and LCN2/NGAL (*p* = 0.007) were also significantly upregulated in fatal outcome (Fig. 4C).

To determine the relationships between the significantly altered factors in fatal vs. survival infection outcomes, correlation analyses were performed (Fig. 4D, E). In the fatal group **(**Fig. 4D**)**, neutrophils were negatively correlated with B cells, MHCII expression by monocytes, and platelets. Resistin, IL-6, and myoglobin were all positively correlated with each other in fatal infection. In the survival group (Fig. 4E**)**, monocytes were negatively correlated with the endothelial marker VCAM-1 and platelets. Monocyte MHCII was also significantly negatively correlated with neutrophils. Taken together, these results demonstrate key immune and endothelial markers that differ depending on the outcome of severe infection. Neutrophilia, low MHCII expression by monocytes, and elevated plasma Resistin, IL-6, myoglobin, and VCAM-1 correlated with fatal infections.

### Immune and endothelial changes are sustained following recovery from moderate COVID-19 infection and correlate with long COVID

**Fig. 5:**
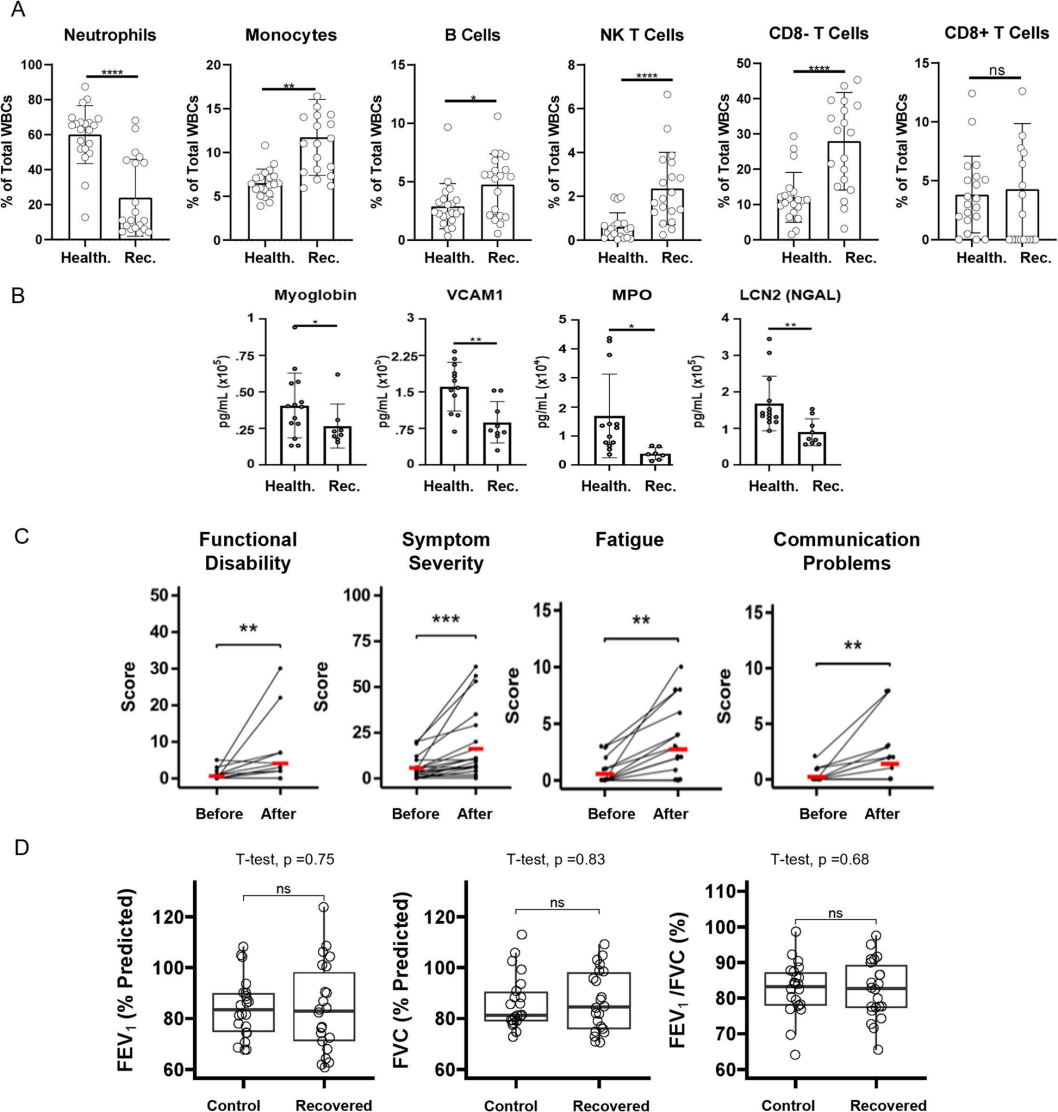
Immune analysis following recovery from infection including long COVID parameters and lung function tests. **A** Quantification of immune populations from healthy control and moderate recovered flow cytometry data (*n* = 18/group, significant outliers removed from analysis). **B** Circulating endothelial damage markers from plasma of control and recovered groups (“Healthy” *n* = 14, “Recovered” *n* = 9). **C** Correlation of self-reported COVID-19 symptoms associated with long COVID as assessed by YRS questionnaire (*n* = 23); points are slightly jittered for visibility. **D** Results of lung spirometry tests measuring overall lung function. Statistical significance determined via unpaired student’s t-test

Given the serious and poorly understood complication of long COVID development after infection, we examined prolonged immune and vascular changes associated with long COVID in recovered individuals in Riverside County and investigate the impact on lung health and immune homeostasis. We were unable to re-recruit individuals from the severe, ICU patient group due to high mortality and declination to participate in the follow-up study. Flow cytometric analysis revealed long-lasting alterations in both innate and adaptive immune cells despite resolution of infection (Supplemental Fig. 5A and Fig. 5A). Notable differences were observed in the frequencies of neutrophils, monocytes, B cells, NK T cells, CD8- T cells, and CD8 + T cells after recovery from moderate infection (Supplemental Fig. 5A). The frequency of neutrophils (*p* < 0.0001) decreased significantly in recovered participants compared to healthy controls (Fig. 5A). Monocytes (*p* = 0.002), B cells (*p* = 0.02), NK T cells (*p* < 0.0001), and CD8- T cells (*p* < 0.0001) remained upregulated following recovery while CD8 + T cells were not changed (*p* = 0.74) (Fig. 5A). These alterations in both innate and adaptive immune populations were sustained as long as 11 months post-positive COVID-19 test and indicate prolonged effects of COVID-19 infection on immune homeostasis.

Circulating cytokines returned to baseline levels in recovered participants (data not shown), but there were endothelial damage indicators that were altered following recovery from infection. Myoglobin (*p* = 0.02), VCAM1 (*p* = 0.002), MPO (*p* = 0.03), and LCN2 (NGAL) (*p* = 0.008) were all significantly downregulated in the recovered group compared to healthy controls (Fig. 5B**)**. These results demonstrate long-lasting effects of COVID-19 infection that could impact endothelial health.

Prolonged changes in the expression of immune and endothelial biomarkers following recovery suggested long COVID presence. To test this, all recovered participants completed the COVID-19 Yorkshire Rehabilitation Scale (YRS) questionnaire for self-reporting of the presence and severity of symptoms defining long COVID. The average reported “Functional Disability” score was significantly increased following COVID-19 infection in the recovered group (Fig. 5C). This score encompasses symptoms related to communication problems, mobility, personal care, daily activity, and social role. The most highly impacted function was communication (adj. *p* = 0.04). There was also a significant increase in “Overall Symptom Severity” after COVID-19 infection. This score encompassed several different physical symptoms such as breathlessness, cough, swallowing, fatigue, continence, pain, cognition, and other psychological symptoms. The most highly impacted symptom was fatigue (adj. *P* = 0.012), but several other symptoms were also significant prior to correction for multiple family-wise comparisons (Table 2).

**Table 2:** Self-reported symptoms as obtained by YRS Questionnaire results

	**Scores**			
Symptom	**Pre-COVID-19**	**Post-COVID-19**	**P**	***P* Adj**
*Breathlessness (at rest)*	0.83 ± 2.61	1.30 ± 2.91	0.05	0.29
*Breathlessness (while dressing)*	1.00 ± 2.86	1.70 ± 3.10	0.06	0.29
*Breathlessness (stairs)*	2.04 ± 2.70	2.65 ± 2.64	0.12	0.49
*Cough or throat symptoms*	0.26 ± 0.62	1.52 ± 2.10	0.01	0.07
*Swallowing problems*	0.04 ± 0.21	0.30 ± 1.11	0.37	0.74
*Fatigue*	0.57 ± 1.08	2.74 ± 3.18	0.00	0.01
*Continence*	0.09 ± 0.42	0.74 ± 2.00	0.17	0.52
*Pain and discomfort*	0.43 ± 0.90	1.96 ± 3.34	0.01	0.11
*Cognition*	0.30 ± 0.70	2.04 ± 2.85	0.01	0.07
*Anxiety*	1.30 ± 1.58	2.48 ± 2.94	0.01	0.08
*Depression*	0.43 ± 0.84	1.30 ± 2.38	0.04	0.25
*PTSD*	0.00 ± 0.00	0.13 ± 0.63	1.00	1.00
*Communication problems*	0.22 ± 0.52	1.39 ± 2.37	0.01	0.04
*Mobility problems*	0.04 ± 0.21	0.70 ± 2.03	0.17	0.52
*Personal care*	0.09 ± 0.43	0.23 ± 0.75	0.59	0.59
*Daily activity problems*	0.17 ± 0.39	1.26 ± 2.14	0.01	0.06
*Social role*	0.09 ± 0.42	0.48 ± 1.20	0.17	0.52

*P-values generated using One-Way ANOVA with multiple comparisons*

The symptoms “Fatigue” and “Communication Problems” were significantly altered after infection following multiple comparison corrections (Fig. 5C). Other symptoms that were significantly altered following recovery from infection were “Breathlessness,” “Cough/Throat Sensitivity,” “Pain,” “Daily Activity Problems,” “Cognition Problems,” “Anxiety,” and “Depression” (Supplemental Fig. 5B). When overall lung health was examined using spirometry testing, lung health was not significantly altered in the recovered group compared to healthy controls indicating a return to baseline lung health (Fig. 5D). In summary, these results demonstrate sustained immunological and endothelial changes long after recovery from infection that suggest prolonged effects of COVID-19 on overall immune health and correlate with the presence of long COVID.

### Participant-specific tracking of immune changes over time during and following COVID-19 infection

**Fig. 6:**
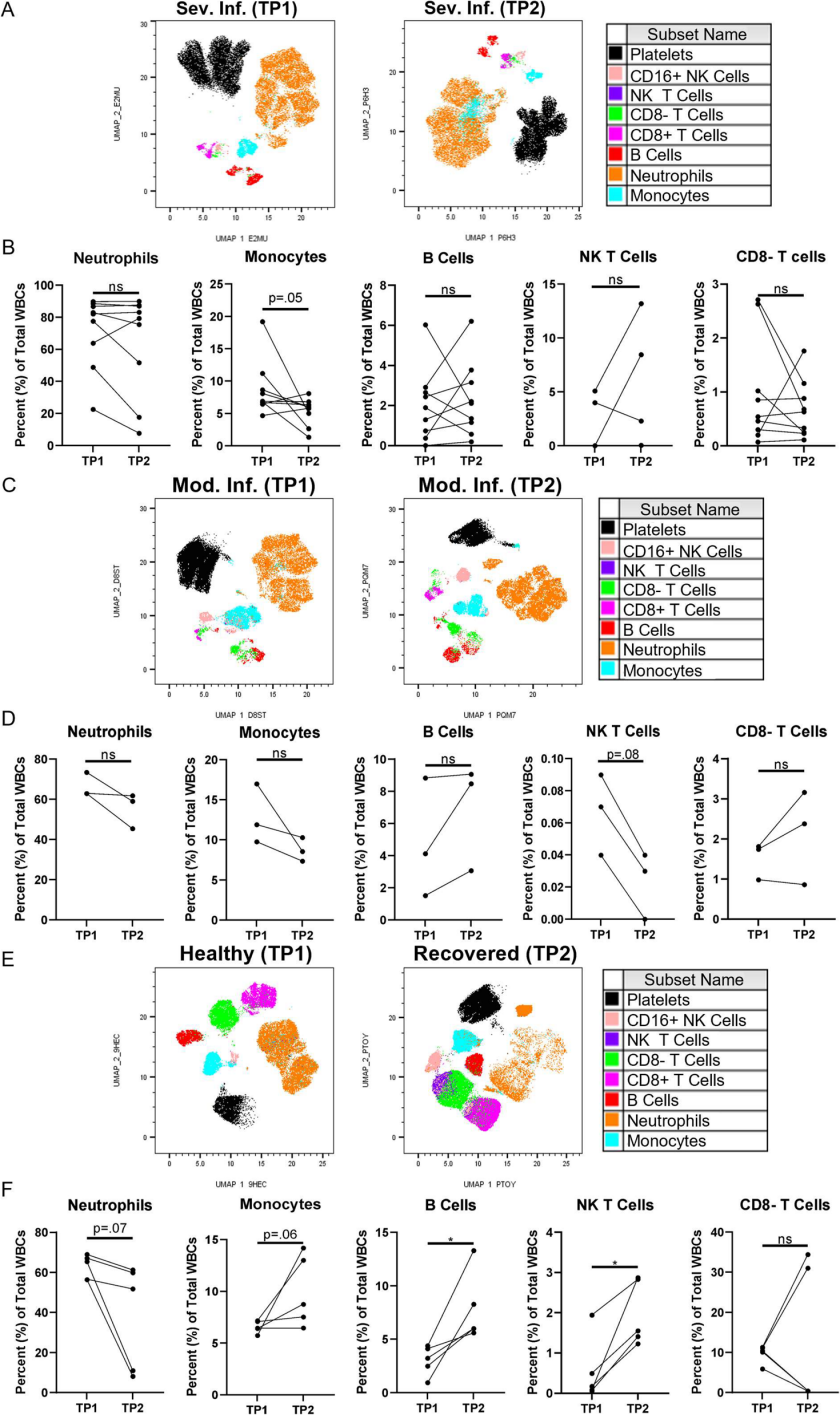
Paired time point analyses of flow cytometry data during severe infection, moderate infection, and following recovery. UMAP analyses (**A**, **C**, and **E**) and graphical quantification (**B**, **D**, and **F**) of flow data from single participants with paired time points for different stages of infection: **A**-**B**) severe infection at 1 day (TP1) and 3 days (TP2) post admission; **C**-**D**) moderate infection at monoclonal antibody treatment start date (TP1) and 7 days aftertreatment start (TP2); E–F) prior to COVID-19 infection (TP1) and following recovery ( varying number of days post COVID + test result) (TP2). Statistical significance determined via unpaired student’s t-test; severe (*n* = 9), moderate (*n* = 3), and control/recovered (*n* = 5) participants at time point one (TP1) and time point two (TP2)

Tracking of immune composition within individual participants at different time points (TP1 and TP2) was performed (Fig. 6). UMAP projections of flow cytometry data enabled the visualization and quantification of immune subsets in severely infected participants at enrollment in the ICU (TP1) and three days later (TP2) (Fig. 6A, B). Participants with moderate infection were also tracked before (TP1), and after treatment with monoclonal antibody (TP2) (Fig. 6C, D). Last, healthy control subjects who subsequently were infected with COVID then recovered, were analyzed for changes in the peripheral blood immune subsets pre- and post-infection (Fig. 6E, F). While the majority of immune cells present did not change from TP1 to TP2, innate monocytes decreased over time in severe infection (*p *= 0.05). In moderate infection, there were decreasing trends in neutrophils, monocytes, and NK T cells and an increasing trend in B cells (Fig. 6D). Paired tracking from uninfected timepoints and six weeks after recovery demonstrated visible changes in all evaluated immune cell-types following recovery from infection (Fig. 6E). Quantification of these immune cells showed increases in B cells (*p* = 0.02), NK T cells (*p* = 0.02), a trend towards monocyte increase (*p* = 0.06) and a decreasing trend in neutrophils (*p* = 0.07) (Fig. 6F). Taken together, these changes observed between individually paired participants show dynamic changes in immune subsets in infection, and also indicate that COVID infection leads to persistent changes in the peripheral immune subsets.

### Machine learning model identifies predictive markers of infection outcome in severe disease

**Fig. 7:**
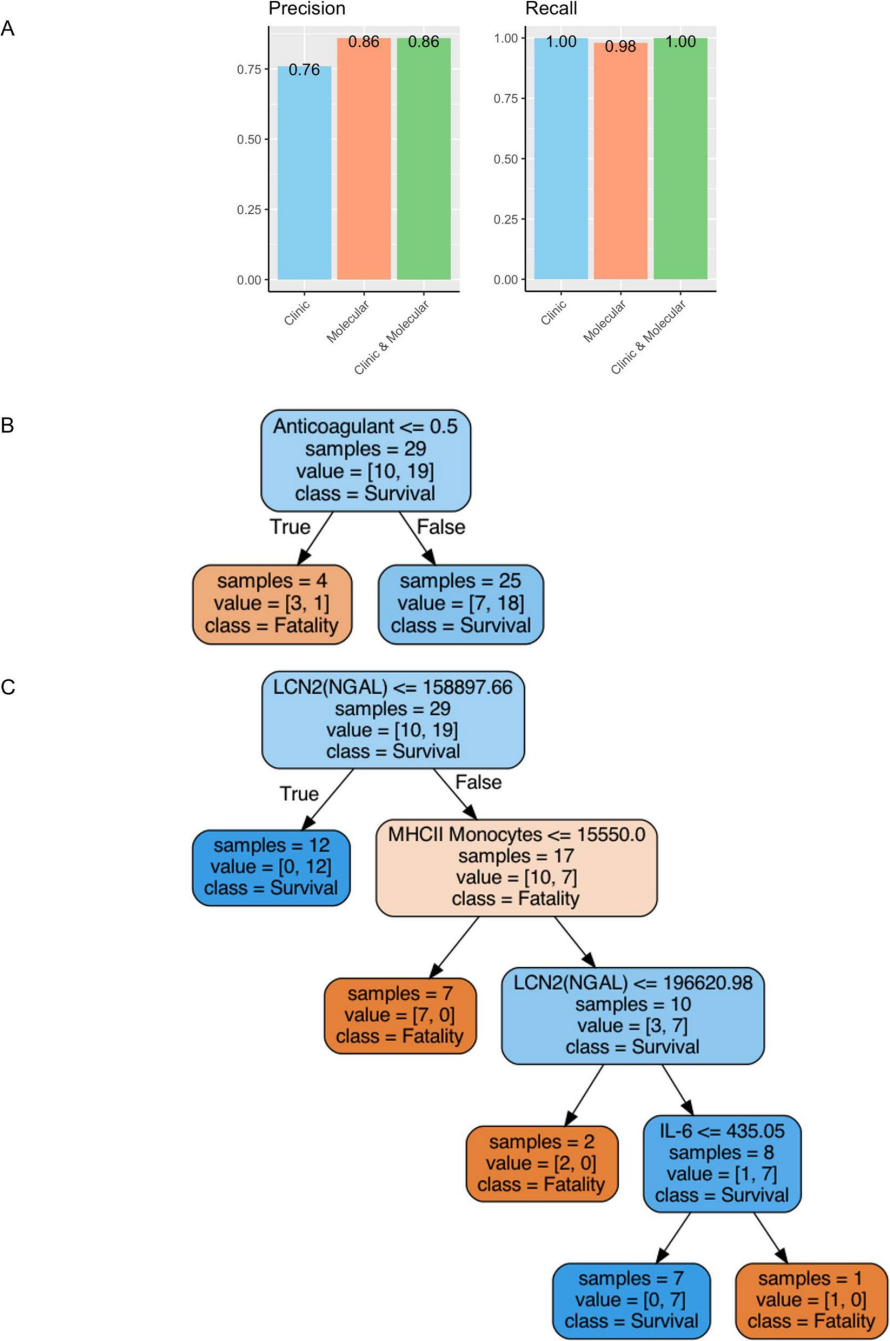
Machine learning analysis. **A** Feature analysis and comparison of our machine learning models across precision and recall metrics predicting survival. Performance of our parameters from the clinic (blue), parameters from molecular (orange) methods vs. parameters from both the clinic and molecular (green) across four metrics. **B**-**C** Decision Tree for Predicting Survival from Severe COVID-19. This figure illustrates the decision tree used to identify significant parameters for predicting survival from severe COVID-19. **B** The tree is first divided based on whether a patient had Anticoagulation treatment. **C** The tree is further divided based on patients’ “LCN2(NGAL),” “MHCII Monocytes,” and “IL-6” levels. With different thresholds from Anticoagulation, LCN2, MHCII Monocytes, and IL-6, the branches lead to different outcomes. The figure also includes the threshold for each parameter, represented by the numbers at the end of each branch, and the sample size from under the threshold from the parameter. Additionally, the figure includes the value which represents the number of possible outcomes of a decision and the decision made

We employed a random forest algorithm as the machine learning model to determine the optimal combination of parameters for predicting survival in severe COVID-19 patients. The metrics mainly investigated were precision and recall. Precision and recall are two of the most relevant metrics to consider when predicting patient survival using machine learning models because they directly measure the ability of the model to correctly identify true positive cases, which are the patients who survive. The results indicated that when utilizing solely parameters obtained from the clinic, the strongest predictor of fatality was [‘Diabetes’, ‘Hispanic’] with a precision of 76% and recall at 100%. When utilizing only parameters from lab-based assays for immune cells and circulating cytokines and endothelial factors, the optimal combination of predictors was ‘'LCN2(NGAL)’',‘'MHCII Monocyte’',‘'IL-6’'], which achieved a precision of 86% and recall at 98%. Notably, when combining all parameters in a single random forest model, the strongest predictors for survival were ‘'LCN2’',‘'MHCII Monocyte’',‘'IL-6’',‘'Anticoagulant’'], resulting in a precision of 86% and recall at 100% (Fig. 7A). This data suggests the strength of combining data collected from the clinic with lab-based assays for improved prediction of survival outcomes in severe COVID-19 patients.

The decision tree analysis using the selected features, namely LCN2, MHCII Monocytes, IL-6, and Anticoagulant, revealed distinct patterns associated with patient survival and fatality outcomes (Fig. 7B, C). In patients receiving anticoagulant therapy (Anticoagulant = 1), there was a notable tendency towards survival, with 72% (18 out of 25) of the patients surviving compared to 28% (7 out of 25) with fatal outcomes (Fig. 7B). In contrast, in the absence of anticoagulant treatment (Anticoagulant = 0), a higher fatality rate of 75% (3 out of 4) was observed, as opposed to a 25% (1 out of 4) survival rate. Furthermore, patients with LCN2 levels below 159 ng/mL exhibited a higher likelihood of survival, as demonstrated by the *n* = 12 in this category (Fig. 7C). Among these, patients with monocytes expressing MHCII at a mean fluorescence intensity lower than 15,550 showed a higher fatality rate, as evidenced by *n* = 7 in this group. In contrast, patients with LCN2 levels above 197 ng/mL had a higher risk of fatality, as indicated by n = 2 in this category. Among patients with LCN2 levels below 197 ng/mL, a lower amount of circulating IL-6 (< 435 pg/mL) was indicative of a higher survival rate, with all 7 individuals surviving (Fig. 7C). This suggests that lower LCN2 and IL-6 levels might be associated with improved prognosis in this patient population. In summary, the decision tree analysis highlights the potential predictive value of LCN2, MHCII expression by monocytes, IL-6, and Anticoagulant treatment in determining survival and fatality outcomes in patients within our study cohort and warrants further investigation in larger cohorts to validate these findings.

## Discussion

### Study overview and novelty

There are currently alarming health disparities such as inadequate access to healthcare among Hispanic communities, especially among those with pre-existing health conditions. These inequities place individuals at a higher risk of severe or fatal disease as shown by previous work [6, 37]. Determining the impacts of COVID-19 infection within at-heightened-risk populations is of vital importance. As a result, the goal of this study was to determine links between immunological and endothelial changes and COVID-19 disease severity in Riverside County, CA, a predominantly high-risk and Hispanic community. Results from this study suggest a higher prevalence of severe disease in individuals of Hispanic ethnicity, which may be driven by increased rates of key clinical co-morbidities, and other sociodemographic factors that impact disease risk and outcomes.

This study is unique as it describes immune and vascular biomarkers associated with disease severity in a high-risk population. It also evaluates the relationship between immune and vascular alterations, long COVID, and lung health which have not previously been demonstrated. The biological relevance of our findings is demonstrated through paired tracking of individual participants’ immune responses at multiple timepoints during infection and after recovery. A machine learning model was used to learn the features that predict patient survival from severe COVID-19 infection and has the potential to uncover hidden insights that might otherwise be overlooked [38].

### Health disparities and severity of COVID-19 infection

This study revealed that a significant proportion of individuals suffering from severe COVID-19 infection in Riverside County, CA, are Hispanic, and these participants report higher instances of co-morbidities, particularly diabetes, hypertension, and obesity. These results highlight likely healthcare disparities in preventative healthcare access in the severe group. According to the RUHS, 84% of their payors use Medi-Cal insurance [39]. Statewide Medi-Cal enrollment data shows that 59% of those patients enrolled are Hispanic/Latino youth, which is more than double the enrollment of Asian/Pacific Islander and White ethnicities [40]. Furthermore, the Riverside area is home to a high number of uninsured individuals [41]. It has been reported that Medi-Cal patients with co-morbidities requiring specialist visits have difficulties in accessing specialist care (i.e., endocrinologists for those with diabetes or cardiologists for those with hypertension). According to the U.S. Bureau of Labor Statistics 2021 data, the average Hispanic/Latino median household income was $55,321 compared to $74,912 for non-Hispanic white households [42]. In 2020, the U.S. Census Bureau reported that 17% of Hispanic individuals were living at poverty level [43]. As a result, few insurance options are available in these cases, and a large percentage of underserved populations utilize Medi-Cal, where they experience difficulties accessing routine preventative care and specialist visits, potentially leading to the worse outcomes seen in the severe infection cohort. Furthermore, limited healthcare access, fear of financial burden, or avoidance of the healthcare system due to documented status can result in patients waiting longer to seek care and therefore receiving treatment only after the disease has advanced to a more severe stage, also leading to increased mortality risk [44].

Previous work has investigated the impact of health disparities on socioeconomic status and overall psychological health as a result of the pandemic [4, 6, 32, 45]. However, it is unknown how health disparities that are prevalent in underserved Hispanic communities relate to infection-dependent biological alterations and infection outcome. The severe infection group in this study was predominantly composed of Hispanic individuals, and this group demonstrated significant changes in immune cell populations and vascular factors which corresponded to a positive correlation with disease severity. As diabetes has been directly linked to increased COVID-19 disease severity [46], the increased frequency of diabetes, which often leads to complications with viral infections, reported by Hispanic individuals in the severe group offers a prime example of health disparities negatively impacting COVID-19 disease outcomes. Based on these cumulative data, further studies are needed to determine the impact of health disparities on the risk of Hispanic communities developing long COVID.

### Severity-dependent changes in immune and endothelial factors and their predictive capability

Several immune factors including both innate and adaptive cell populations are necessary to successfully combat COVID-19 infection [8, 14, 18, 30, 47–49]. The severe infection group showed significant alterations to all immune subsets compared to healthy controls and moderate infection, indicating relationships between immune parameters and disease severity. Neutrophils and the neutrophil chemoattractant IL-8, which are known to cause increased damage during COVID-19 infection, were significantly increased in this group compared to both healthy controls and the moderate infection group [8, 9, 11, 12]. In our global correlation analyses, neutrophils were also positively correlated with the inflammatory cytokine IL-6, and all of these immune parameters were positively correlated with severe infection. These same factors and Resistin, which has been identified as an indicator of disease severity and early predictor of mortality in sepsis [35], were also increased in individuals who had a fatal infection outcome. This data demonstrates a direct link between neutrophils, neutrophil-chemotactic biomarkers, and disease severity in our high-risk study cohort. Contrary to neutrophil patterns observed, all other immune subsets and Type I interferons were significantly decreased in the severe group. These immune parameters were also negatively correlated with severe infection and positively correlated with healthy participants in our correlation analyses. These opposing immune populations in healthy versus severe infection supports the importance of these cells for combatting infection as previously reported [15, 18, 30, 48]. Lack of these cells in the severe group could indicate an impaired antiviral response.

Measurement of endothelial biomarkers showed severity-dependent changes in circulating vascular damage indicators. The severe infection group demonstrated significantly elevated circulating levels of myoglobin, VCAM-1, and MPO which were significantly correlated with severe infection. MPO and VCAM-1 were also positively correlated with Hispanic ethnicity in this group, and myoglobin was significantly elevated in fatal infection. Myoglobin has been directly linked to oxidative vascular damage [50–52], which could explain increased lung pathology in fatal COVID-19 infection. The elevated levels of MPO seen directly correspond to the observed increases in neutrophils in severe infection, and MPO production by neutrophils has been associated with COVID-19 disease pathology [8]. In addition, MPO showed significant positive correlations with Resistin and IL-6 in fatal infection. Currently, techniques are being developed to create potential drug targets for MPO [53] and myoglobin [54] in COVID-19 disease that could be used to mitigate COVID-19 disease severity caused by vascular damage.

Our machine learning results demonstrate that the inclusion of lab-based assays in addition to routine assays performed in the clinic can significantly improve the accuracy of the predictive models, indicating the complementary nature of the two types of parameters investigated in this study. We found that ‘LCN2(NGAL),’ ‘MHCII expression by monocytes,’ ‘IL-6,’ and ‘Anticoagulant treatment’ revealed distinct patterns associated with severe infection outcomes. In severe COVID-19 patients receiving anticoagulant therapy, a tendency towards survival was observed, matching previous studies that have reported the potential benefits of anticoagulant treatment in this patient population [55, 56]. Emerging evidence from our study also suggests that lower LCN2 levels might be associated with improved prognosis in severe COVID-19 patients. LCN2, or Lipocalin-2 (also known as neutrophil gelatinase-associated lipocalin or NGAL), is an acute-phase protein involved in immune response and inflammation [57]. Recent work has indicated that elevated LCN2 levels are correlated with the severity of COVID-19 infection and may serve as a potential biomarker for disease progression [58]. Our finding that higher MHCII expression by monocytes is associated with improved prognosis confirms results found in recent studies [59, 60]. The increased expression of MHCII by monocytes in severe COVID-19 patients that survive infection suggests that a more robust immune response contributes to better clinical outcomes and resistance to re-infection [61]. Recent studies have indicated that severe COVID-19 patients with lower inflammatory interleukin-6 (IL-6) levels may exhibit improved prognosis [62, 63]. This association between the combination of anticoagulant therapy, lower LCN2 levels, increased expression of MHCII by monocytes, lower IL-6 levels, and improved prognosis warrants further investigation as it could aid in developing targeted therapies and personalized treatment strategies for COVID-19 patients. In the context of healthcare, even modest improvements in predictive accuracy can have significant impacts on patients' lives, informing treatment decisions and resource allocation [64]. By leveraging machine learning to analyze these limited datasets, researchers can gain valuable insights into disease mechanisms, prognosis, and therapeutic approaches, ultimately improving patient care and outcomes.

### Recovery from infection and biomarkers for long COVID

Previous studies have identified sustained immune alterations following recovery from mild and severe COVID-19 infection [25, 26, 48]. In addition, the presence of long COVID and subsequent immune changes have been investigated previously [24, 28, 29]. Recent studies reported that women, black individuals, and Hispanic individuals are more likely to experience long COVID [65]. Consistent with these studies and the primarily Hispanic makeup of Riverside County, our recovered group was composed of 43% Hispanic individuals which accounted for more than any other single ethnicity.

Our work shows significant increases in monocytes, B cells, NK T cells, and CD8- T cells following recovery from mild-to-moderate infection as well as significant decreases in neutrophils and circulating vascular biomarkers compared to healthy controls. This indicates a prolonged systemic inflammatory response despite successful clearance of active infection in these recovered participants, which has been hypothesized with previous work demonstrating increased immune activation. These results were also seen across our paired timepoint analyses of healthy controls who later returned as recovered participants. As a result, our data suggests potential risks of autoimmunity and/or overactive immune responses following COVID-19 infection.

Our study adds to current knowledge of long-term COVID-19 impacts by showing previously undemonstrated prolonged vascular alterations. We also offer insights into the impact of prior COVID-19 infection on lung health via the use of spirometry measurements. Recent work has identified specific immune signatures that underly certain post-acute COVID-19 sequalae in the lung linking prolonged immune dysregulation with lasting tissue pathology caused by infection [20]. This indicates the potential for sustained immune alterations to have deleterious effects on overall lung health following recovery from infection and during long COVID. Our results indicate that despite sustained immune changes and self-reported symptoms suggesting the presence of long COVID, overall lung function is recovered as no significant change was observed in spirometry tests between our recovered and healthy control participants.

### Limitations and caveats

While our study offers novel insights into the long-term health impacts of COVID-19 infection, there remain limitations. First, there was missing information for various participants in each group, such as missing blood collection for some participants due to logistic issues. Second, different treatment regimens offered and given to severe and moderate infection groups (such as Remesdivir/dexamethasone for severe group, monoclonal antibody treatment for moderate group, and Paxlovid for certain recovered participants) could have affected the immune and/or vascular endothelial responses observed. Third, our modest sample size, including our paired participant time point data, limits our ability to generalize these results, and increased participant numbers are needed to perform more rigorous statistical analysis in the future. The addition of increased data representing the Hispanic population in the future could help with comparisons of Hispanic versus non-Hispanic groups and discern population-specific immune alterations. There are also age differences between the Moderate/Severe infection groups and the Healthy Control/Recovered groups which could contribute in part to differences observed in certain biomarkers. The sample size for the Yorkshire Rehabilitation Scale Questionnaire is also limited. Despite this smaller sample size, our study agrees with several other studies worldwide that utilize this questionnaire [66–69]. Lastly, it should be considered that the immune response is extremely complex and can vary greatly between individuals which could contribute to some of the variation seen in the results of our study. In addition, our machine learning model made use of a small sample size that warrants further testing to confirm validity of our results for this portion of our study. Further prospective clinical studies that address these caveats are needed to better understand the relationships between COVID-19 disease severity and immune changes as well as the relationship between long COVID and sustained immune alterations.

### Supplementary Information

**Additional file 1: Fig. S1.** Confirmation of active COVID-19 infection for severe and moderate infectiongroups. A) Quantification of viral burden from nasal swab of severe and moderate infection groups. B) Quantification of blood spike protein in severely infected participants based on survival vs fatality from infection. **Fig. S2.** Full gating strategy for whole blood flow cytometry. Concatenated healthy control sample used as in Figure 2. **Fig. S3.** Flow plots of significantly altered immune cell types by study group. A-C) Flow plots of major immune cell populations analyzed for control (A), moderate infection (B), and severe infection (C) groups (all individual samples concatenated by group for shown plots). **Fig. S4.** Correlation matrices of demographic, immune, and endothelial parameters in severe COVID-19 infection with fatal and non-fatal outcomes. A) Fatal outcome correlation matrix. B) Survival outcome correlation matrix. **Fig. S5.** Significantly altered immune cell types and long COVID symptoms in Recovered group. A) Flow plots of major immune cell populations analyzed for moderate recovered group (all individual samples concatenated by group for shown plots). **B**) Correlation of self-reported COVID-19 symptoms associated with long COVID identified as significantly altered prior to multiple comparison corrections as assessed by YRS questionnaire (*n*=23); points are slightly jittered for visibility.

## Data Availability

The datasets used and/or analyzed during the current study are available from the corresponding author on reasonable request.
