# Supplementary material for: Health disparities in COVID-19: immune and vascular changes are linked to disease severity and persist in a high-risk population in Riverside County, California
Source: BMC Public Health. 2023 Aug 19;23:1584. doi: 10.1186/s12889-023-16462-5 (PMC10439554; doi:10.1186/s12889-023-16462-5)

Supplemental Figure 1: Confirmation of active COVID-19 infection for severe and moderate infection groups. A) Quantification of viral burden from nasal swab of severe and moderate infection groups. B) Quantification of blood spike protein in severely infected participants based on survival vs fatality from infection.

Supplemental Figure 2: Full gating strategy for whole blood flow cytometry. Concatenated healthy control sample used as in Figure 2.

Supplemental Figure 3. Flow plots of significantly altered immune cell types by study group. A-C) Flow plots of major immune cell populations analyzed for control(A), moderate infection (B), and severe infection (C) groups (all individual samples concatenated by group for shown plots).

Supplemental Figure 4: Correlation matrices of demographic, immune, and endothelial parameters in severe COVID-19 infection with fatal and non-fatal outcomes. A) Fatal outcome correlation matrix. B) Survival outcome correlation matrix.

Supplemental Figure 5. Significantly altered immune cell types and long COVID symptoms in Recovered group. A) Flow plots of major immune cell populations analyzed for moderate recovered group (all individual samples concatenated by group for shown plots). B) Correlation of self-reported COVID-19 symptoms associated with long COVID identified as significantly altered prior to multiple comparison corrections as assessed by YRS questionnaire (n=23); points are slightly jittered for visibility.

Supplemental Figure 1

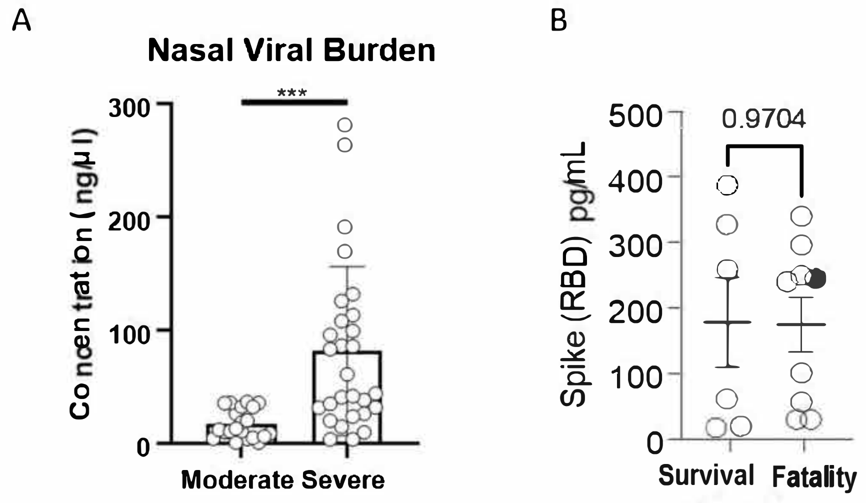

Supplemental Figure 2

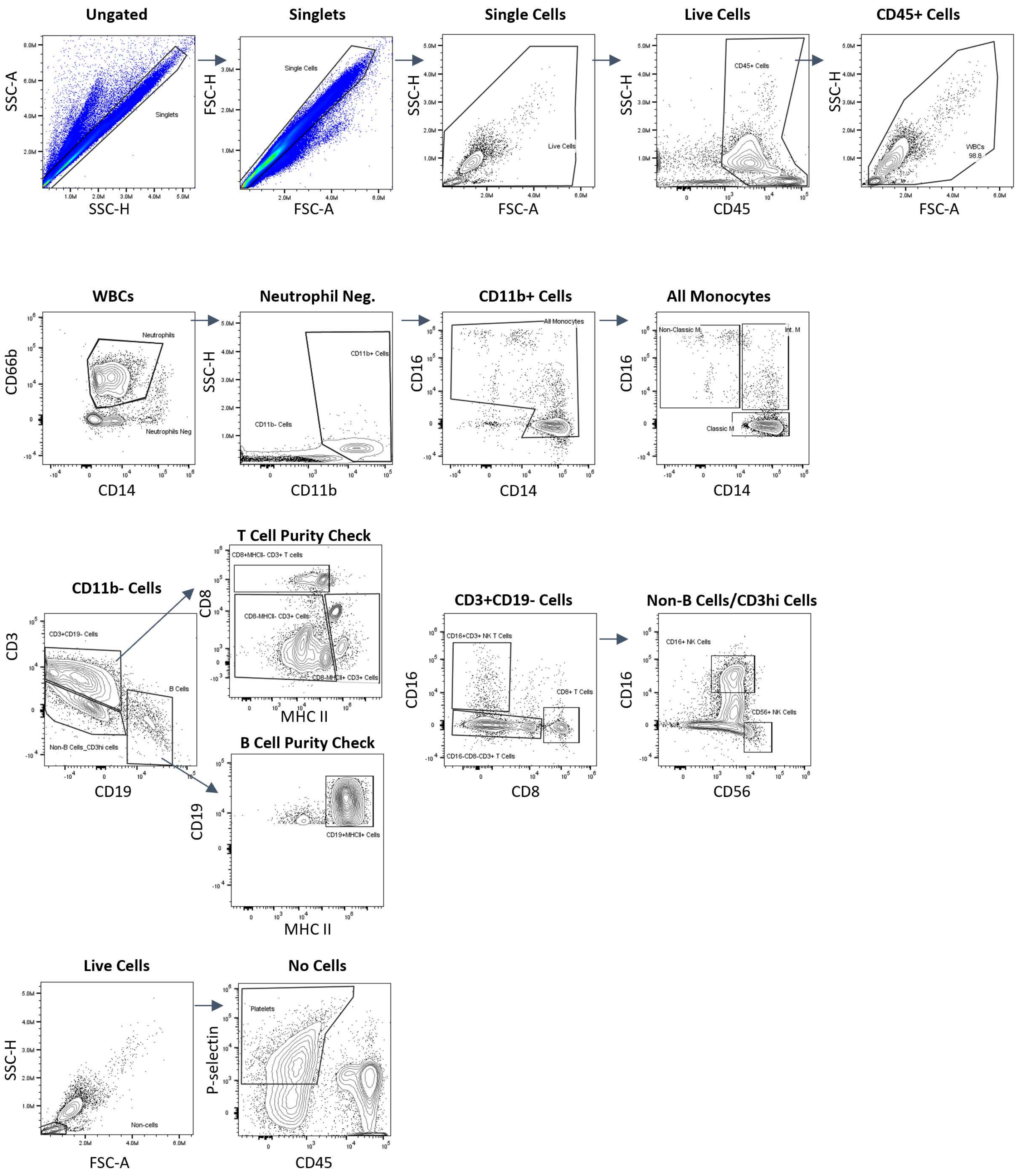

Supplemental Figure 3

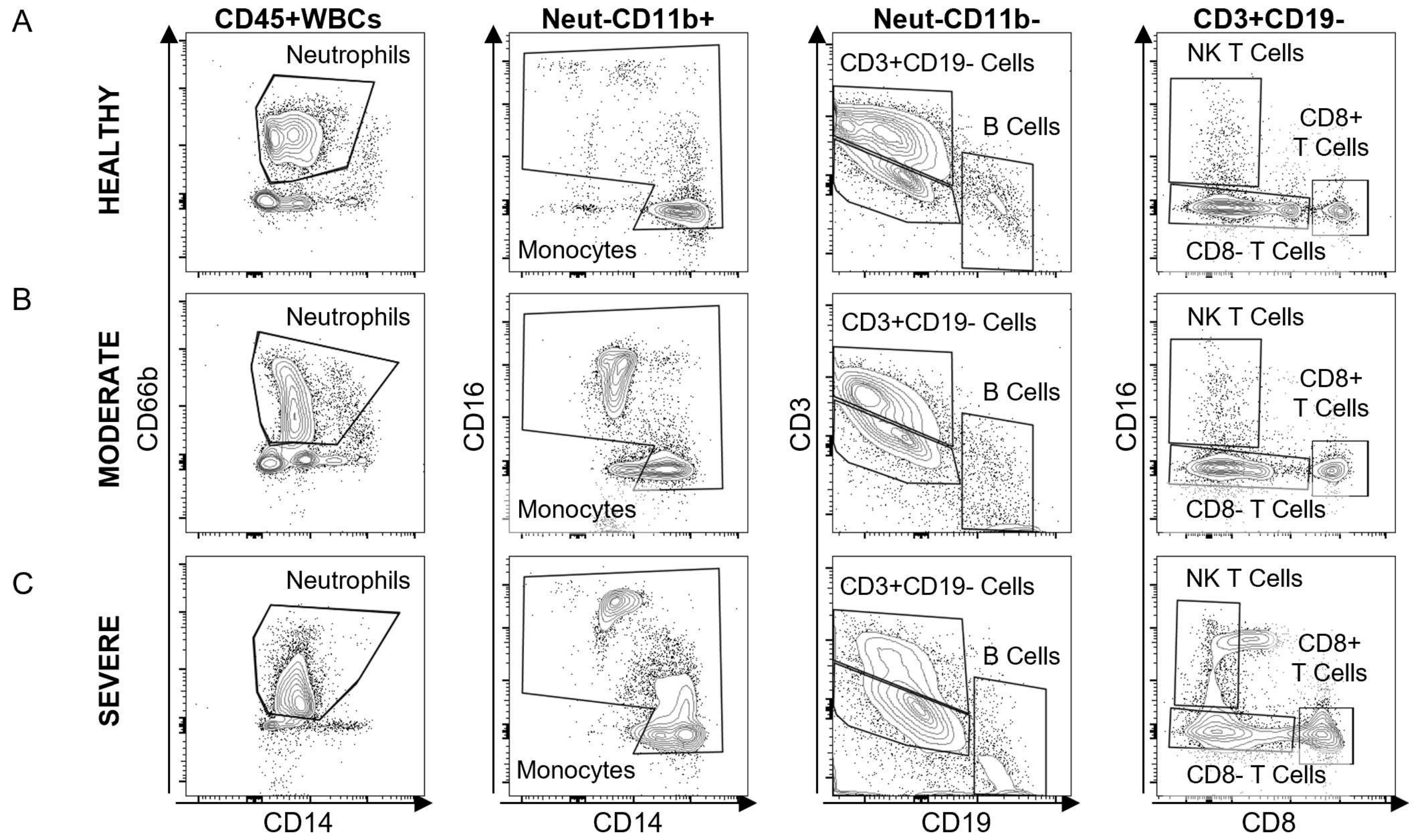

Supplemental Figure 4

A

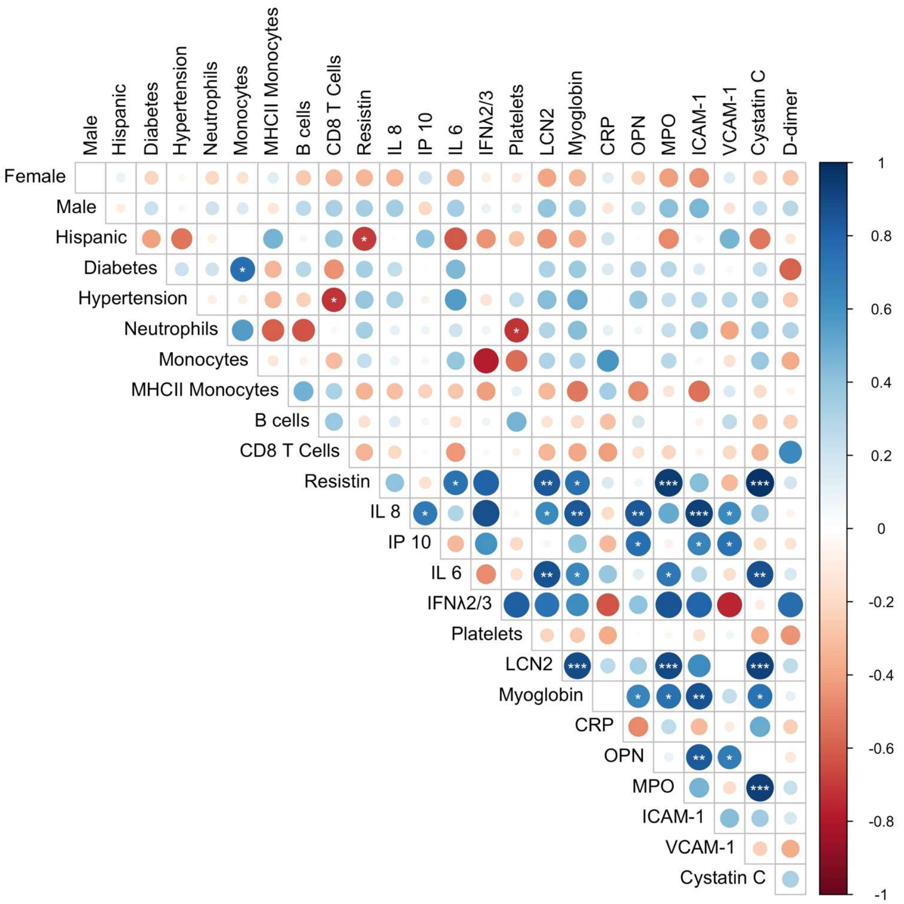

B

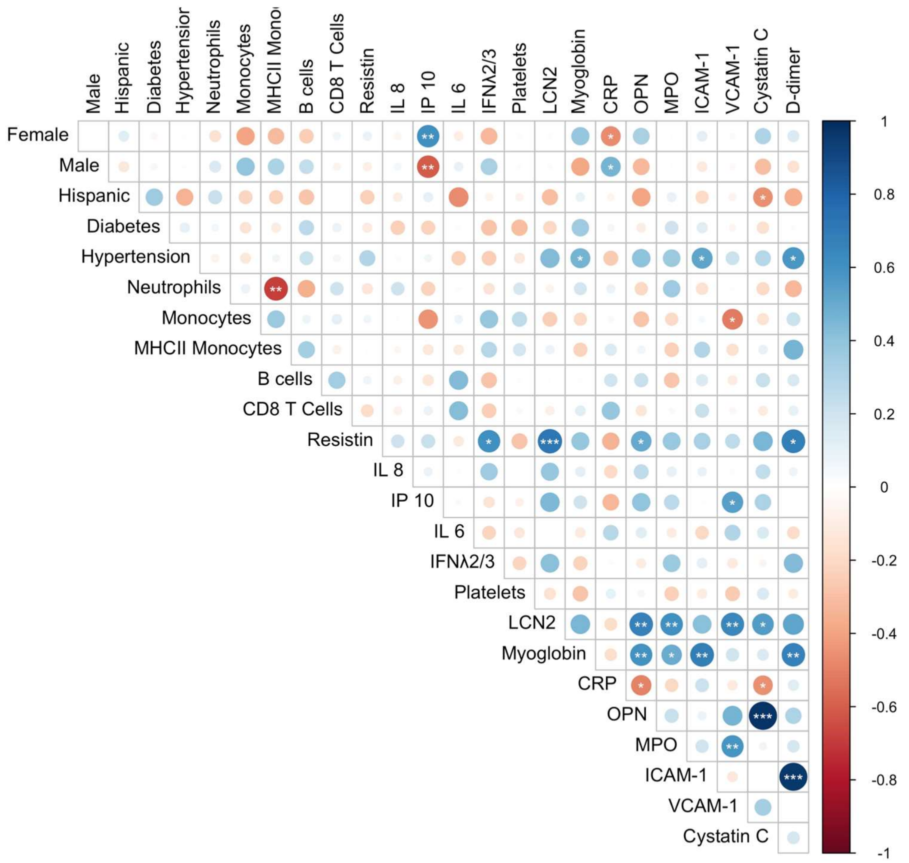

Supplemental Figure 5

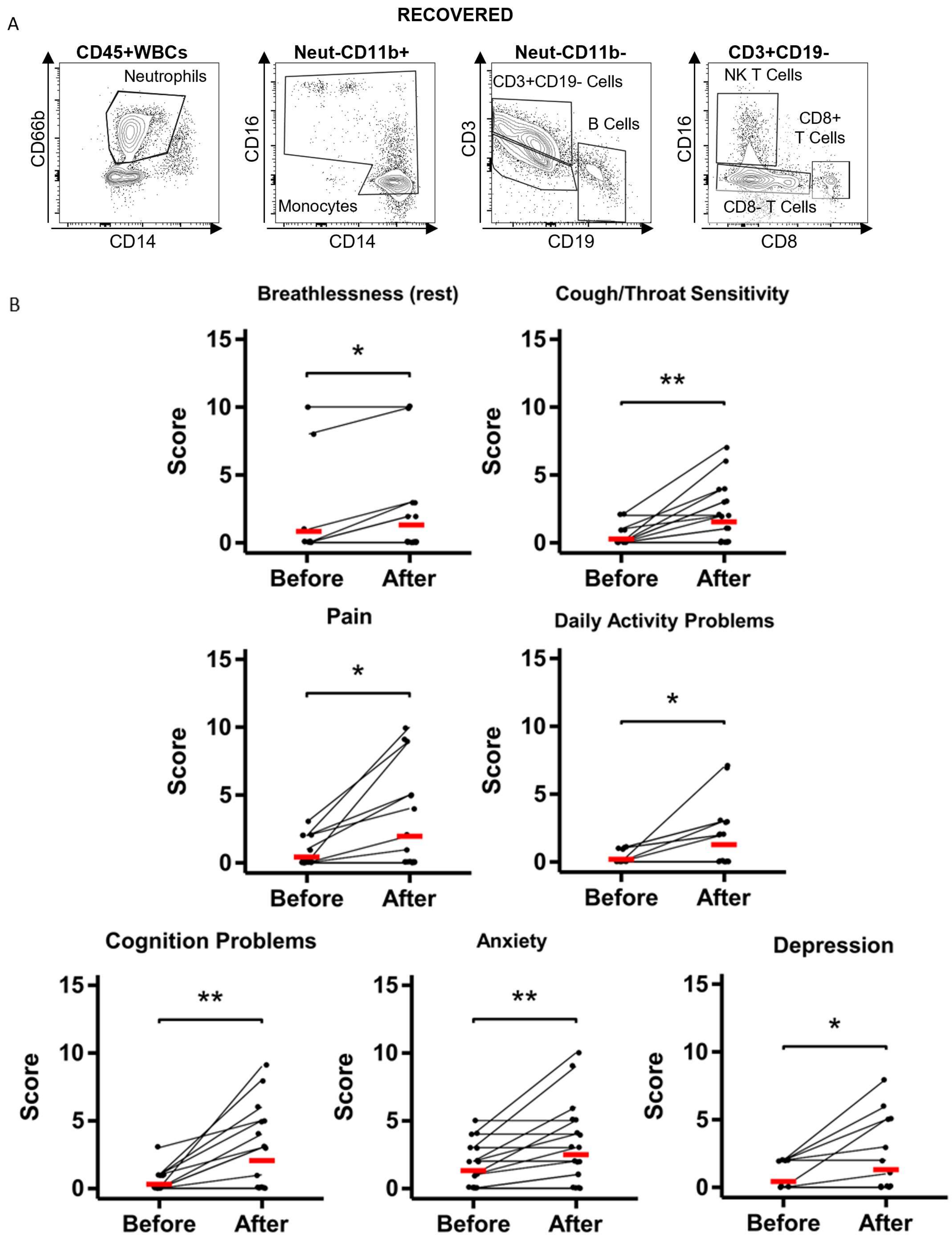

Supplement: Supplementary file 1 — Additional file 1: Fig. S1. Confirmation of active COVID-19 infection for severe and moderate infectiongroups. A) Quantification of viral burden from nasal swab of severe and moderate infection groups. B) Quantification of blood spike protein in severely infected participants based on survival vs fatality from infection. Fig. S2. Full gating strategy for whole blood flow cytometry. Concatenated healthy control sample used as in Figure 2. Fig. S3. Flow plots of significantly altered immune cell types by study group. A-C) Flow plots of major immune cell populations analyzed for control (A), moderate infection (B), and severe infection (C) groups (all individual samples concatenated by group for shown plots). Fig. S4. Correlation matrices of demographic, immune, and endothelial parameters in severe COVID-19 infection with fatal and non-fatal outcomes. A) Fatal outcome correlation matrix. B) Survival outcome correlation matrix. Fig. S5. Significantly altered immune cell types and long COVID symptoms in Recovered group. A) Flow plots of major immune cell populations analyzed for moderate recovered group (all individual samples concatenated by group for shown plots). B) Correlation of self-reported COVID-19 symptoms associated with long COVID identified as significantly altered prior to multiple comparison corrections as assessed by YRS questionnaire (n=23); points are slightly jittered for visibility. [file 12889_2023_16462_MOESM1_ESM.pdf]
